# Old and New NICE Guidelines for the Evaluation of New Onset Stable Chest Pain: A Real World Perspective

**DOI:** 10.1155/2018/3762305

**Published:** 2018-11-08

**Authors:** Nazario Carrabba, Angela Migliorini, Silvia Pradella, Manlio Acquafresca, Marco Guglielmo, Andrea Baggiano, Giuseppe Moscogiuri, Renato Valenti

**Affiliations:** ^1^Cardiovascular and Thoracic Department of Careggi Hospital, Florence, Italy; ^2^Radiologic Department of Careggi Hospital, Florence, Italy; ^3^Centro Cardiologico Monzino, IRCCS, Milan, Italy

## Abstract

Stable chest pain is a common clinical presentation that often requires further investigation using noninvasive or invasive testing, resulting in a resource-consuming problem worldwide. At onset of 2016, the National Institute for Health and Care Excellence (NICE) published an update on its guideline on chest pain. Three key changes to the 2010 version were provided by the new NICE guideline. First, the new guideline recommends that the previously proposed pretest probability risk score should no longer be used. Second, they also recommend that a calcium score of zero should no longer be used to rule out coronary artery disease (CAD) in patients with low pretest probability. Third, the new guideline recommends that all patients with new onset chest pain should be investigated with a coronary computed tomographic angiography (CTA) as a first-line investigation. However, in real world the impact of implementation of CTA for the evaluation of new onset chest pain remains to be evaluated, especially regarding its cost effectiveness. The aim of the present report was to discuss the results of the studies supporting new NICE guideline and its comparison with European and US guidelines.

## 1. Introduction

In the last two years a great debate occurs about the value of anatomical information by coronary computed tomographic angiography (CTA) in comparison to functional imaging tests on the evaluation of patients with new onset chest pain and unknown coronary artery disease (CAD) [1–5]. Stable chest pain is a common clinical presentation that often requires further investigation using noninvasive or invasive testing [6–8]. Recently, PROMISE [9] and SCOT-HEART [10] studies suggest that an evaluation strategy based on CTA improves diagnostic certainty, as well as efficiency of triage to invasive catheterization; it also may reduce radiation exposure when compared with functional stress testing, with similar rates of cardiac events. Moreover, the EVINCI [11] trial supports the use of CTA for stable chest pain, highlighting a better performance in comparison to other imaging strategies. After the publication of these studies, the National Institute for Health and Care Excellence (NICE) [12] recommended CTA as the first-line investigation for all patients presenting with chest pain due to suspected CAD.

## 2. Functional and Anatomical Tests for Suspicion of CAD

In current era of modern cardiology, the diagnostic workup test for patients with suspicion of CAD remains matter of debate [6–8]. Whatever the use of functional or anatomical tests, their additional values should be implied on guide the decision-making process to improve outcome, reducing cardiac death and nonfatal myocardial infarction [13–21]. For this purpose, it is conceivable that the first-line diagnostic test should have a high level of diagnostic accuracy as well as the ability to better stratify individuals risk and, finally, the ability to establish proper treatment regimes. In addition, taking into account a reduced economical resource of the health system around the world, in the practice the cost-effective clinical aspects may play a pivotal role in planning a diagnostic workup test for CAD. Several studies have compared different stress imaging modalities in order to detect obstructive CAD. However, there are no strict recommendations based on the evidence of one diagnostic test' superiority over another [9–21]. Specifically, diagnostic functional tests are encumbered by a high rate of false-positive results. The low prevalence of obstructive CAD following elective ICA has been clearly demonstrated in the registry data, raising more criticisms regarding the ability of functional test in detecting markers of significant myocardial ischemia [22, 23]. All this has generated a great debate as to which test is best placed to serve as a “gatekeeper” to invasive coronary angiography (ICA). The rationale for the use of noninvasive testing prior to ICA is established in the recent publication of Clinical Evaluation of Magnetic Resonance Imaging in Coronary Artery Disease 2 (CE-MARC-2), confirming that risk models may overestimate the presence of obstructive CAD [21]. Ideally, considering several noninvasive tests available, ICA is only rarely mandatory to confirm the diagnosis of obstructive CAD and should be reserved for those likely to have coronary intervention. In the last decade, the additional values of coronary CTA to improve diagnostic accuracy and risk stratification of coronary artery disease have been evaluated in depth from several studies. Recently, two ‘test-and-treat' multicenter randomized control trials furnished evidences into whether coronary CTA could be incorporated into chest pain care pathways [9, 10]. In similar way, both trials were focused on the evaluation if the incorporation of noninvasive test into a care pathway may confer benefit to patients with suspicion of CAD. Namely, the Prospective Multicenter Imaging Study for Evaluation of chest pain (PROMISE) trial enrolls a large cohort from USA and Canadian centers in order to settle whether an initial assessment of suspected stable CAD using CTA improves outcomes, reducing major adverse cardiovascular events [9]. This study demonstrated that coronary CTA was associated with more ICA within the first 90 days; however the use of coronary CTA reduced invasive angiograms without obstructive CAD. Moreover, at 2-year follow-up, the use of CTA was not associated with improvement in death, myocardial infarction, or major procedural complication in comparison to functional strategy. Differently, the Scottish Computed Tomography of the HEART (SCOT-HEART) trial recruited UK patients referred for recent onset chest pain to cardiology clinics with suspected angina [10], all of whom presented with chest pain and one-third reported typical angina symptoms. In comparison with a PROMISE trial, a higher rate of obstructive CAD was reported in CTA arm of SCOT-HEART. Moreover, there was a nonsignificant reduction in cardiac death and myocardial infarction (hazard ratio 0.62, 95% confidence interval 0.38–1.01, p=0.0527). In the SCOT-HEART study, differently from the PROMISE study, coronary CTA did not replace functional testing but was added to a standard care protocol with exercise ECG for most. In these two studies the low prevalence of CAD and the low occurrence of MACE in patients with stable chest pain were reported, raising questions concerning the use for new imaging test. Generally, it is well known that, in patients with low CAD prevalence, the probability of CAD may be overestimated by standard prediction rules [9, 10]. Indeed, the prevalence of CAD in PROMISE was low 8.8% in comparison to the 53% predicted probability by the Diamond and Forrester model. In the SCOT-HEART study, a higher rate of obstructive CAD was reported in the CTA arm compared with the PROMISE trial. Moreover, in the SCOT-HEART study, after 50 days clinicians reviewed the test result and started preventive medical therapy. From this time, post hoc landmark analysis was associated with an impressive reduction rate of cardiac death and myocardial infarction (hazard ratio 0.50, 95% confidence interval 0.28–0.88, p=0.020) [23]. Thus, not surprisingly, the findings of randomized SCOT-HEART study confirmed the results previously reported from the observational CONFIRM registry, where for the first time the beneficial effect of statin therapy in individuals with subclinical atherosclerosis was demonstrated [24]. Importantly, the longer-term impact of coronary CTA use in clinical practice remains unexplored. Until recently, the long-term clinical outcomes of the SCOT-HEART trial was published [25], showing that the use of CTA in comparison to than standard care alone is associated with a lower rate of death or nonfatal myocardial infarction (2.3% versus 3.9%; HR 0.59; 95% CI 0.41 to 0.84; P = 0.004). These results are mainly related to the change of treatment based on CTA findings. In addition, the use of CTA is associated with an increase rate of ICA in the short follow-up, but 5 years the use of ICA and coronary revascularization were not different. Moreover, according to design of study, the SCOT-HEART trial encouraged the secondary prevention strategy in patients with nonobstructive CAD. This strategy may be very important, considering that near the half of subsequent myocardial infarctions occurred among patients with nonobstructive CAD. The clinical, social, and financial implications of this consideration could be very relevant in the next future, considering the exponential increase of the subclinical and nonobstructive coronary atherosclerosis reported from CTA in patients with suspicion of CAD.

## 3. NICE Guidelines Update 2016

Specifically, the NICE guideline update (2016) makes three key changes to the 2010 version [12]. The first is the recommendation for a clinical assessment of the likelihood of CAD, based on the typicality of the chest pain into typical, atypical, or noncardiac, instead of the previous pretest probability (PTP) risk score (RS). The second change in the guideline is that a zero calcium score is no longer used to rule out CAD in patients with low PTP. Thirdly, and most radically, NICE now recommends that all patients with new onset chest pain with atypical or typical anginal features, as well as those with noncardiac chest pain and an abnormal resting ECG, should first be investigated with CTA using a 64-slice (or above) CT scanner. Functional imaging tests are now reserved for the assessment of patients with chest pain symptoms who are known to have CAD and for patients where the CTA has been nondiagnostic or has shown CAD of uncertain significance.

## 4. Comparison with European and American Recommendations

The 2013 European Society of Cardiology (ESC) guideline on stable chest pain recommends the use of a PTP RS that is calculated using age, gender, and typicality of chest pain, but not cardiovascular risk factors [26]. This RS is based on an updated Diamond-Forrester method, which adjusts the likelihood of CAD for a more contemporary population [26]. The ESC recommends that patients with an intermediate RS (15–85%) have a functional imaging test and, if there is limited availability, exercise ECG is recommended as an alternative in patients with RS 15–65% and CTA for patients with RS 15–50%. The 2012 guidelines from the American cardiology societies on stable chest pain recommend clinical evaluation of the PTP of CAD [27]. Patients able to exercise with interpretable resting ECGs and a low to intermediate likelihood of CAD are recommended to have an exercise ECG. Patients with uninterpretable ECG and patients with intermediate to high likelihood of CAD are recommended to have functional imaging tests. Patients with low to intermediate PTP, who are unable to exercise, may also undergo CTA as an alternative to exercise stress testing.

## 5. The 2016 Update to NICE CG95 Guideline

The PTP model was based on USA cohorts of patients undergoing invasive coronary angiography (ICA) in the 1970s who had a much higher prevalence of CAD than current rapid access chest pain clinic populations. Thus, in the latest guidance, NICE has parted from its PTP model; it may overestimate the risk in current rapid access chest pain clinic populations [28, 29]. Since the ESC RS was based on a contemporary population and has been externally validated and shown to be a good predictor of risk [29, 30], there was an expectation that NICE may adopt it. Not surprisingly, the most striking change in the new NICE guideline is the expansion of the use of CTA to all patients with new onset of chest pain. NICE no longer recommends coronary artery calcium scoring followed by CTA if the calcium score is above zero because of case reports of significant coronary stenoses in patients with a zero calcium score. Another reason is that the radiation dose from CTA on high-specification CT scanners is now as low as the radiation dose for the calcium score itself (less than 1 mSv). More controversially, NICE expanded the recommendation for CTA as first line to patients with intermediate and high likelihood of CAD based on their cost-effectiveness analysis suggesting that this would be a lower cost strategy. While recent clinical trials, such as PROMISE, demonstrated that patients investigated with CTA and functional tests had similar clinical outcomes [9], one has to remember that this trial was in a low-intermediate disease prevalence population with only approximately 11% having CAD. Although the SCOT-HEART trial had a higher prevalence of CAD and demonstrated that the use of CTA in patients with chest pain improves the diagnosis when added to standard of care, the standard of care was the exercise ECG and not functional imaging tests [10]. In fact, there are no published data demonstrating the diagnostic accuracy or cost effectiveness of CTA in patients with chest pain and higher likelihood of CAD, making the NICE recommendations for CTA in this population somewhat surprising. Interestingly, however, there are UK data demonstrating higher utilization rates of the costly ICA following a CTA strategy [31].

## 6. Disinvestment in Stress Imaging Services in Favor of CT Imaging: A New Question

The advance in management and the adoption of modern effective treatment have reduced the cardiovascular mortality for patients with acute coronary syndrome worldwide, but not for patients with stable CAD. Currently, healthcare system focused their efforts on delivering management of stable CAD that is both clinical and cost effective. Notably, the populations with stable CAD increase in age and consequently their access to the healthcare system may increase exponentially worldwide. This picture of stable CAD is true worldwide; thus the treatment for stable CAD should be not only efficacious but also sustainable for healthcare system. Moreover, differently to the previous two decade, now the rapid clinical assessment of patients with suspected angina is necessary for the community of cardiology to select out high-risk individuals [32]. In this way, the attending physician may avoid the risk of potential complications following the onset of chest pain symptoms, as well as may avoid unnecessary diagnostic tests. In current clinical practice, the ICA remains the more precise test to confirm or to exclude the presence of obstructive CAD against which all other noninvasive tests have been validated. Since the hospitalization is required to perform ICA, this examination remains the most expensive diagnostic investigation and, importantly, it exposes individuals to the highest risk of procedural complications, although the radial access may reduce the burden of complications and now should be the preferred approach [33]. After a number of publications, now coronary CTA is recognized as accurate diagnostic test to evaluate the presence of coronary atherosclerosis. The publication of the updated NICE guideline CG95 confirms and reinforces this message, but in the same time it rises some concerns about the impact of the use of coronary CTA as first-line investigation on the resource of healthcare system. In England the adoption of this strategy for CAD diagnosis has been estimated as favorable, since it will be associated with an annual savings of £16 million, by prompt exclusion of significant CAD and more effectively use of resources [34]. However, caution needs in interpretation these findings, since the availability of CT scan in other countries may be different in comparison to the UK, and the applicability of this model in other healthcare system remains to be explored. On this regard, the British Society of Cardiovascular Imaging/British Society of Cardiovascular CT (BSCI/BSCCT) estimated an impressive increase of near 700% in coronary CTA across the UK [35]. The situation is quite similar in most European countries as well as in USA, although the UK has a relatively low number of CTA scanners per head of population [35]. Thus, the potential adoption for most western countries of updated NICE recommendations for stable CAD may require a substantial investment in CTA technology and training. Out of the setting of randomized and well conducted trial, in clinical practice the use of CTA will generate a number of equivocal CTA test, due to the nonoptimal expertise in exam execution and image interpretation. Consequently, in order to solve the doubt raise from the equivocal tests the attending physician may require in the diagnostic workup more additional functional imaging test. Thus, it is likely that the potential disinvestment in stress imaging services in favor of CTA imaging could not occur in the next years. Notably, the rapid development of new CT technologies may help us to reduce the burden of false-positive CTA for patients with stable CAD. In this setting, the routine use of fractional flow reserve CTA and CT perfusion (CTP) appears to be the most promising and valuable tool, even if the accuracy of both techniques remains to be demonstrated on revascularized patients with recurrent chest pain [36–38]. In addition the availability of both modern techniques is very restricted in some dedicated centers. Moreover, in case of wide dissemination of coronary CTA in western countries, the preservation of quality imaging is very important, since it is strongly related to the diagnostic accuracy of coronary CTA. In this regard, current recommendation should be followed in order to optimize image quality in cardiac CTA [39, 40], and, moreover, standardized informative reports of CTA studies should be provided by the physician as well as the radiation dose exposure should be reported on this report. Recently, CAD-RADS reporting and data system are also available for the structured reporting of cardiac CTA, which may facilitate and simplify the communication of results to clinicians and patients [41]. Especially, this program may optimize downstream investigations in order to avoid an increase in the use of ICA when cardiac CTA identifies nonobstructive CAD. Not surprisingly, in setting of chest pain patients with suspicion of CAD the nonobstructive CAD was the most common pattern of coronary atherosclerosis disease and its detection may increase with the increase availability and use of coronary CTA. Currently, the management of nonobstructive CAD represents in the community of cardiology one of the major challenge. The concept of stenosis severity alone for the classification of CAD appears old and it does not feet with the continuum of risk associated with nonobstructive atherosclerotic plaque. The identification of features of vulnerability in coronary plaques rather than the luminal narrowing in isolation may improve the risk stratification of future cardiac events [42–56]. This concept is very important, especially in women with suspicion of CAD showing frequently a nonobstructive CAD or minimal coronary lesion from CTA, associated with features of microvascular dysfunction [57].

## 7. The Radiation Dose

The practical implementation of the new guidelines will meet many challenges. The recommendations are in part based on the assumption that the radiation dose of CTA is in the order of 1–2 mSv, which is achievable in most patients with the latest generation CT scanners [58–62]. However, most UK hospitals do not have these and instead use 64-slice CT scanners that can perform a CTA with a radiation dose of 3–5 mSv, provided prospective gating is used, which requires a heart rate of 60 bpm; otherwise, CTA is performed with retrospective gating, which allows for a heart rate up to 70 bpm, but with radiation doses of 10–15 mSv, a similar radiation dose to MPS. In PROSPECT, a trial comparing CTA and MPS in patients with intermediate risk chest pain, the total radiation dose was high in both arms (24 versus 29 mSv) and no difference was found in the rates of ICA between the two strategies. To implement the NICE guidelines without increasing the radiation burden on the population, the National Health Service (NHS) will need to make a significant investment in high-specification CT scanners and/or carefully consider the choice of the follow on functional imaging test, based on the patient's age, sex, and their cumulative radiation dose, from other radiation-based investigations. This UK picture is similar to that observed in the majority of the European country, highlighting that the investment in the latest generation CT scanners should be planned as an appropriate and rationale strategy rather than occasional investment. A further major challenge, if the NICE recommendations are to be adopted on the next European guidelines, will be to identify and train the increased requirement for radiographers and consultants (both cardiologist and radiologist) to perform and report the additional CTA.

## 8. Conclusions

CTA is an excellent rule-out test for CAD when used in the appropriate disease prevalence population. Despite the good performance of CTA in recent PROMISE, SCOT-HEART, and EVINCI trials, there is little clinical or health economic data to support the use of CTA over other noninvasive imaging tests in patients at intermediate-high risk of CAD in real world. Furthermore, the availability of latest generation scanners in European country is limited. Thus the potential for high cumulative radiation dose exposure from multiple serial CTA investigations could be a big problem. Finally, in real world the implementation of CTA for the evaluation of new onset chest pain fundamentally depends on the new health strategy based on the reconfiguration of current finances and staffing levels.
