# Corrigendum to “Old and New NICE Guidelines for the Evaluation of New Onset Stable Chest Pain: A Real World Perspective”

**DOI:** 10.1155/2019/9637490

**Published:** 2019-03-07

**Authors:** Nazario Carrabba, Angela Migliorini, Silvia Pradella, Manlio Acquafresca, Marco Guglielmo, Andrea Baggiano, Giuseppe Muscogiuri, Renato Valenti

**Affiliations:** ^1^Cardiovascular and Thoracic Department of Careggi Hospital, Florence, Italy; ^2^Radiologic Department of Careggi Hospital, Florence, Italy; ^3^Centro Cardiologico Monzino, IRCCS, Milan, Italy

In the article titled “Old and New NICE Guidelines for the Evaluation of New Onset Stable Chest Pain: A Real World Perspective” [1], the last name of the seventh author was given incorrectly as Moscogiuri. The author's name should have been written as Muscogiuri. The revised authors' list is shown above.
